# Proteostasis Failure in Neurodegenerative Diseases: Focus on Oxidative Stress

**DOI:** 10.1155/2020/5497046

**Published:** 2020-03-27

**Authors:** Annika Höhn, Antonella Tramutola, Roberta Cascella

**Affiliations:** ^1^Department of Molecular Toxicology, German Institute of Human Nutrition Potsdam-Rehbruecke (DIfE), Nuthetal, Germany; ^2^German Center for Diabetes Research (DZD), Muenchen, Neuherberg, Germany; ^3^Department of Biochemical Sciences, Sapienza University of Rome, Rome, Italy; ^4^Department of Experimental and Clinical Biomedical Sciences, Section of Biochemistry, University of Florence, Florence, Italy

## Abstract

Protein homeostasis or proteostasis is an essential balance of cellular protein levels mediated through an extensive network of biochemical pathways that regulate different steps of the protein quality control, from the synthesis to the degradation. All proteins in a cell continuously turn over, contributing to development, differentiation, and aging. Due to the multiple interactions and connections of proteostasis pathways, exposure to stress conditions may cause various types of protein damage, altering cellular homeostasis and disrupting the entire network with additional cellular stress. Furthermore, protein misfolding and/or alterations during protein synthesis results in inactive or toxic proteins, which may overload the degradation mechanisms. The maintenance of a balanced proteome, preventing the formation of impaired proteins, is accomplished by two major catabolic routes: the ubiquitin proteasomal system (UPS) and the autophagy-lysosomal system. The proteostasis network is particularly important in nondividing, long-lived cells, such as neurons, as its failure is implicated with the development of neurodegenerative diseases, such as Alzheimer's disease, Parkinson's disease, and amyotrophic lateral sclerosis. These neurological disorders share common risk factors such as aging, oxidative stress, environmental stress, and protein dysfunction, all of which alter cellular proteostasis, suggesting that general mechanisms controlling proteostasis may underlay the etiology of these diseases. In this review, we describe the major pathways of cellular proteostasis and discuss how their disruption contributes to the onset and progression of neurodegenerative diseases, focusing on the role of oxidative stress.

## 1. Proteostasis

Protein homeostasis or “proteostasis” is the process that regulates the homeostasis of the intracellular pool of functional and “healthy” proteins. The cellular protein quality control ensures the proper folding of newly synthesized proteins, handling unfolding, refolding, and/or degradation of misfolded proteins [1]. This process is critical as 30% of newly synthesized proteins are prone to misfolding [2]. Proteostasis becomes even more important for those nondividing cells such as neurons, whose proteostatic machineries are reduced with aging, causing an accumulation of damaged organelles and misfolded proteins [3, 4]. The two main cellular degradation systems are the ubiquitin proteasomal system (UPS), which is responsible for degradation of both functional and dysfunctional proteins, and the autophagy-lysosomal system that degrades whole organelles, large aggregates of proteins/macromolecules, and single proteins.

### 1.1. Protein Folding, Refolding, and Misfolding

The mechanism that governs the folding of proteins is a complex phenomenon of biomolecular self-assembly resulting in the “energy landscape” theory [5]. To ensure efficient folding and prevent aggregation, cells express some classes of molecular chaperones that guide the nascent polypeptide chain along a productive folding pathway, avoiding and sometime reversing misfolding and aggregation [6]. As proteins are structurally dynamic and suffer the external and endogenous stress, it is essential that the chaperones also cooperate with machineries of protein degradation in a large protein homeostasis (or proteostasis) network [3, 7]. This protein network serves to maintain a balanced proteome. If a protein fails to fold correctly, the cell utilizes extensive security measures to maintain its function. Chaperones will attempt to remedy the unfolded protein first, and if unsuccessful, they are able to activate several different cellular programs, including the unfolded protein response (UPR), heat shock response (HSR), ubiquitin-proteasome system (UPS), and endoplasmic reticulum-associated degradation (ERAD) to take more drastic measures to either fix the problem or destroy the unfolded protein altogether [8]. Protein folding is however intrinsically error-prone because, during the search for the stable native-like contacts between residues, some events that be termed “misfolding” can take place. Even if small proteins may fold rapidly and with full yield *in vitro* [9], folding is often inefficient for larger proteins, owing to off-pathway aggregation. Thus, the failure of proteins to fold or remain folded under physiological conditions represents a problem of great biological and medical importance [6]. The main causes of misfolding and aggregation are nonspecific interactions between exposed hydrophobic regions, which segregate to avoid unfavorable contacts with water and polar/charged moieties. To protect these regions from nonspecific aggregation, many chaperones preferentially bind to hydrophobic regions within unfolded chains, acting as molecular machineries responsible for the quality control of protein folding. Novel functions of chaperones in higher eukaryotes have been shown to be the binding to preformed aggregates to promote their disassembly [10], to mask their hydrophobic patches [11, 12], or to convert them into large assemblies [13, 14], therefore suppressing their toxicity in all three cases.

When the chaperone machinery fails following pathological insults that induce endoplasmic reticulum (ER) stress the unfolded proteins are accumulated in ER, activating the UPR mechanism. The activation of UPR restores ER proteostasis primarily through the transcriptional remodeling of ER protein folding, trafficking, and degradation pathways as UPS system.

Misfolded proteins can originate from two pathways. First, there are proteins that are correctly translated using the amino acid sequences but sometimes find an alternatively stable conformation and thus misfold. Alternatively, genetic mutations may cause protein misfolding and malfunction; even one erroneous amino acid can cause an entire protein to fold incorrectly, and the results may include aggregation of the protein and cellular catastrophe. Beyond the possibility of a genetic mutation in a protein specific to a disease, it is also possible for protein misfolding disorders to arise because of mutations in the cell's protein folding machinery. Mutations in chaperones allow proteins that are properly translated to fold into alternative conformations more freely, while mutations in degradation-related proteins allow misfolded proteins to escape degradation and subsequently aggregate and form fibrils.

Protein misfolding is also associated with cellular insults, such as mitochondrial dysfunction, calcium dysregulation, and inflammation. There are more than 40 human diseases that are directly associated with the deposition of such aggregates in tissue [15], including neurodegenerative diseases such as Alzheimer's disease and Parkinson's disease. The majority of cases is age-dependent, since apparently facilitated through a decline in the capacity of the proteostasis network that occurs during aging [4, 16]. For each of these pathologies, there is a key protein that is repeatedly produced and misfolded, evading both the protein folding machinery and cellular degradation mechanisms, and begins to form aggregates that nucleate out into large fibrillar aggregates [17].

### 1.2. Ubiquitin-Proteasome-System (UPS)

**Figure 1:**
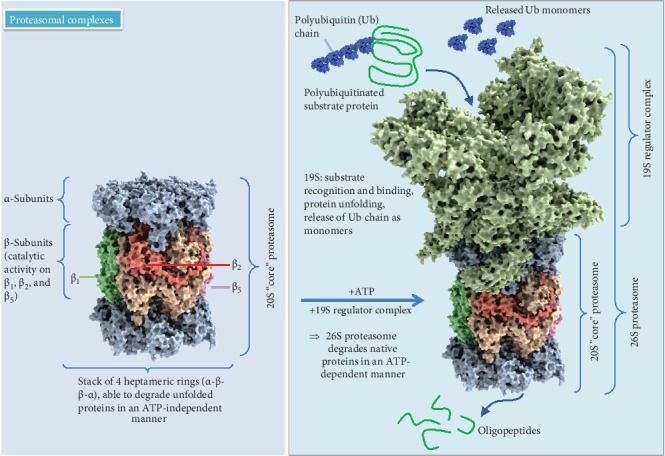
Proteasomal complexes. 20S and 26S models according to data from X-ray structure analysis [267].

The ubiquitin-proteasome system (UPS) is a cardinal proteolytic system targeting the vast majority of intracellular proteins, essential for normal cell function. It consists of ∼850 different components [18–20] and is responsible for the proteolytic degradation of 80–90% of all cellular proteins, including many regulated, short-lived, misfolded/damaged ones, or otherwise obsolete proteins [21]. The 20S proteasome, which is the core particle where the proteolysis takes place, contains four stacked rings that form a barrel-shaped molecule with a central cavity. This structure is the functional core of all proteasomal forms, complemented by regulatory components influencing specificity and activity of the 20S proteasome (Figure 1) [22–24]. These stacked rings include two noncatalytic outer rings called *α*-rings (with subunits *α*1-*α*7) and two catalytic inner rings called *β;*-rings (accordingly *β*1-*β*7). While the *α*-rings are responsible for substrate recognition, binding and admission of proteins to the inner chamber, and binding of regulatory components [25, 26], the three proteolytic activities are limited to the *β*-rings including chymotrypsin-like (*β*5), caspase-like (*β*1), and trypsin-like (*β*2) activity [27]. Association of 19S with the *α*-rings of the core particle results in the formation of 26S proteasomes with one (RP1CP; 19S-20S) or two (RP2CP; 19S-20S-19S) regulatory particles. The 19S particle contains at least 18 subunits, with a base composed of six ATPases exerting a chaperone-like activity and a lid composed of 8 subunits able to recognize polyubiquitin signals, enhancing the spectrum of degraded proteins. The 19S proteasome binds and unfolds ubiquitinated proteins and opens the entry gate of the 20S proteasome to allow protein degradation in the central cavity [27]. To mark proteins for degradation, the covalent labeling of target proteins with ubiquitin, a small protein with 76 amino acids, is required [28]. This conjugation generally involves three different enzymes: E1 (ubiquitin-activating enzyme) hydrolyses ATP and forms a thioester-linked conjugate between itself and ubiquitin; E2 (ubiquitin-conjugating enzyme) receives ubiquitin from E1 and forms a similar thioester intermediate with ubiquitin; and E3 (ubiquitin ligase) binds both E2 and the substrate and transfers the ubiquitin to the substrate [29]. In some circumstances, a fourth ubiquitination enzyme, E4 (ubiquitin chain elongation factor), is required, together with the other three enzimes, to extend a polyubiquitin chain [30]. A minimum of four ubiquitins is necessary for proteasomal targeting of the protein [31]. After polyubiquitination, substrate proteins are transferred to the 26S proteasome, where they are degraded into oligopeptides; furthermore, ubiquitin molecules are released by ubiquitin recycling enzymes and can be reused for new polyubiquitination reactions [32]. Despite its high affinity for polyubiquitinated proteins, the 26S proteasome is poor in degrading oxidized proteins [33–35], which also do not undergo preferential ubiquitination [36–38]. An intact ubiquitination system is actually not required for selective degradation of oxidatively modified proteins [39, 40], since already unfolded proteins can reach the inner proteasomal chamber without the assistance of a regulator particle or ubiquitination. Furthermore, oxidation of proteins may induce a conformational change of the protein structure and expose hydrophobic amino acids to the surface, normally buried in the protein interior. The recognition of these hydrophobic residues is the suggested mechanism of selective proteasomal degradation of oxidatively modified proteins [41]. However, since hydrophobic surfaces of highly oxidized proteins can probably interact with other proteins to form aggregates, this can lead to the inhibition of proteasomal degradation due to aggregate size and structure [42].

### 1.3. Immunoproteasome

Oxidative stress and stimulants such as interferons, tumor necrosis factor-*α*, and lipopolysaccharide [43, 44] can lead to the assembly of the inducible form of the proteasome, also called immunoproteasome, by replacing the constitutively expressed catalytic subunits with the respective immunoproteasomal subunits: low molecular weight protein 2 (LMP2 or *β*1i), multicatalytic endopeptidase complex-like 1 (MECL1 or *β*2i), and the low molecular weight protein 7 (LMP7 or *β*5i) subunit. In addition, 11S (PA28, proteasome activator 28 kDa) regulators can bind to the 20S core unit. The immunoproteasome has primarily been studied for its role in processing oligopeptides for antigen presentation to the adaptive immune response. Beyond its role in the immune system, the immunoproteasome is also of relevance in the clearance of oxidized proteins [45, 46]. As mentioned above, the 26S proteasome is poor in degrading oxidized proteins, and additionally oxidized proteins do not preferentially undergo ubiquitination [35–37]. During oxidative stress, the 26S proteasome disassembles, in turn, increasing the pool of available 20S proteasomes [47]. This promptly increases cellular capacity to degrade oxidized proteins and increases cellular ability to cope with oxidative stress. 11S regulators also show increased binding to 20S proteasomes resulting in an increased capacity to selectively degrade oxidatively damaged proteins following oxidative stress [45, 46].

There is indeed accumulating evidence that the immunoproteasome preferentially degrades oxidized proteins with an activity and selectivity equal to or greater compared to that of the 20S proteasome [29, 45, 46]. Binding of the 11S regulator to the immunoproteasome improves both activity and selectivity for oxidized proteins [45, 46], so it seems to be clear that the immunoproteasome has an expanding biological role in addition to its role in immune function [48–50]. Furthermore, it was shown that cells even react to very low levels of H_2_O_2_ with an increase in 20S, immunoproteasome, and 11S regulator [51] and that protein damage is not necessarily needed for their upregulation. This suggests that mild stimulus activates signal-transduction pathways that prepare the cell to tolerate future oxidative insults [52]. In addition, increased immunoproteasomal function potentially contributes to lifespan differences in mice and among primate species [53]. What selective pressures could have driven enhanced immunoproteasome levels in primates for which a long lifespan is selectively beneficial is unknown, but an enhancement in immunoproteasomal function contributes to an increased cellular proteostatic capacity and turnover of oxidized and damaged proteins.

Expression and activity of the immunoproteasome have been also linked to diseases, such as cancer, inflammatory, and neurodegenerative diseases [52]. Increased expression of the immunoproteasome has been identified within amyloid plaques [54], suggesting that cells try to clear away protein aggregates even if unsuccessful. Immunoproteasome was also found to be increased in astrocytes and microglia of ALS mouse models [55], and inhibition of immunoproteasome induction results in reduced survival [56], proposing that immunoproteasome could play a role in restricting disease severity. It is well accepted that an age-related decrease in proteasomal activity weakens the cellular capacity to remove oxidatively modified proteins and paves the way for the development of diseases [57–60], whereas the enhanced presence of immunoproteasomes in aging brain potentially reflects a sustained defense [61]. Overall, a functional UPS is essential, and any alteration to its components potentially has pathological consequences.

### 1.4. Endoplasmic Reticulum-Associated Protein Degradation (ERAD)

As mentioned above, one task of the proteasome is the degradation of newly synthesized misfolded proteins. Newly synthesized proteins are kept in the endoplasmic reticulum (ER) until their maturation is completed. Correct protein folding is essential for homeostasis, but despite all of the effort dedicated to protein folding, a considerable portion of newly synthesized polypeptides entering the ER fails to acquire a native conformation [62]. ER protein quality control mechanisms are responsible to monitor the precision of protein folding. Proteins that fail proper folding or assembly are subjected to the ER-associated protein degradation (ERAD), responsible to clear the ER from these potentially harmful species. ERAD is a complex, multistep process starting with recognition and targeting of substrates, followed by retrotranslocation, ubiquitination, and proteasomal degradation. Due to this complexity, this quality-control process is only briefly described here, with reference to reviews focusing only on ERAD describing the steps in detail [63, 64]. In general, ERAD substrates can be recognized by maturation tags due to posttranslational modifications, and chaperones and other recognition factors participate in the recruitment of misfolded proteins to the ERAD complex, including BiP/GRP78, EDEM1 (ER degradation enhancing *α*-mannosidase like protein 1) and the lectins OS9 (osteosarcoma amplified 9), and XTP3B [65–68], which can bind to unfolded proteins deliver them to the Sel1L-Hrd1 ERAD complex in preparation for transport to the cytosol. Furthermore, it is clear that de-mannosylation is required for degradation of misfolded glycoproteins, and inhibition of this mannose cutting leads to stabilization of misfolded glycoproteins in the ER [69]. Truncation of terminal mannose from branch C exposes *α* terminal *α*1,6-bonded mannose residue as recognition signal for OS9 and XTP3-B. After being selected, ERAD substrates are retrotranslocated across the ER lipid bilayer for ubiquitination by ER-resident transmembrane E3s and their cognate E2s, followed by extraction from the membrane in an ATP-dependent manner and release into the cytoplasm for proteasomal degradation [63]. Misfolded luminal proteins need to be completely retrotranslocated, whereas for membrane proteins, only the transport of certain domains is required. Consequently, ubiquitination of luminal substrates occurs only at late stages of retrotranslocation, once a portion of the substrate has been exposed to the cytoplasm, whereas ubiquitination of most, but not all [70], membrane substrates is linked to their retrotranslocation [63]. After retrotranslocation from the ER to the cytosol, ERAD substrates need to be rapidly targeted to proteasomal degradation in order to avoid aggregate accumulation in the cytosol. For coupling of retrotranslocation with degradation, ubiquitinated substrates need to be recognized by cytosolic proteins operating as ubiquitin receptors. Ubiquitin-binding domain containing proteins have the ability to shuttle ubiquitinated proteins from the retrotranslocation complex at the ER membrane to the proteasome [71, 72]. Under basal conditions, ERAD functions are at low levels, with increasing activity at proteotoxic stress conditions. Increased ER stress is observed in postmortem brain samples from AD patients, where increased levels of GRP78/BiP and the activation of UPR in the temporal cortex and the hippocampus occur consistently with NFTs and A*β* plaque formation [73].

Accumulation of unfolded or misfolded proteins in the ER lumen triggers ER stress by decreasing free chaperone levels [74], followed by activation of the UPR mechanism and an attenuation of protein synthesis [75] to slow down the entry of newly synthesized proteins to the ER. UPR also enhances ERAD capacity by upregulation of ERAD genes to ensure degradation of defective proteins when folding attempts fail [73].

Finally, under conditions of consistently increasing cellular stress, UPR also induces cell death mechanisms such as apoptosis or autophagy [75].

### 1.5. Autophagy-Lysosome System

Besides the proteasomal pathway, the lysosomal system is the most important cellular “digestive mechanism” with a nearly unlimited degradative capacity. Lysosomes are membrane-enclosed organelles, equipped with a variety of enzymes, including hydrolases, proteases, lipases, peptidases, glycosidases, nucleases, phosphatases, and sulfatases. Lysosomes play an important role in the degradation of extracellular and intracellular macromolecules. Extracellular material can be invaginated via pino-, phago- or receptor-mediated endocytosis. Soluble long-lived proteins, extracellular material, large protein aggregates, and even organelles can be targeted to the lysosome in a process called autophagy. The term autophagy or “self-eating” was coined in 1960s following the observation of double-membrane vesicles entrapping intracellular organelles and proteins [76]. The autophagic system consists of ∼500 different components [18, 20, 77, 78] targeting larger aggregates or membrane-associated proteins [79, 80] and has a key role in cell survival and in preserving cell metabolic balance [81].

**Figure 2:**
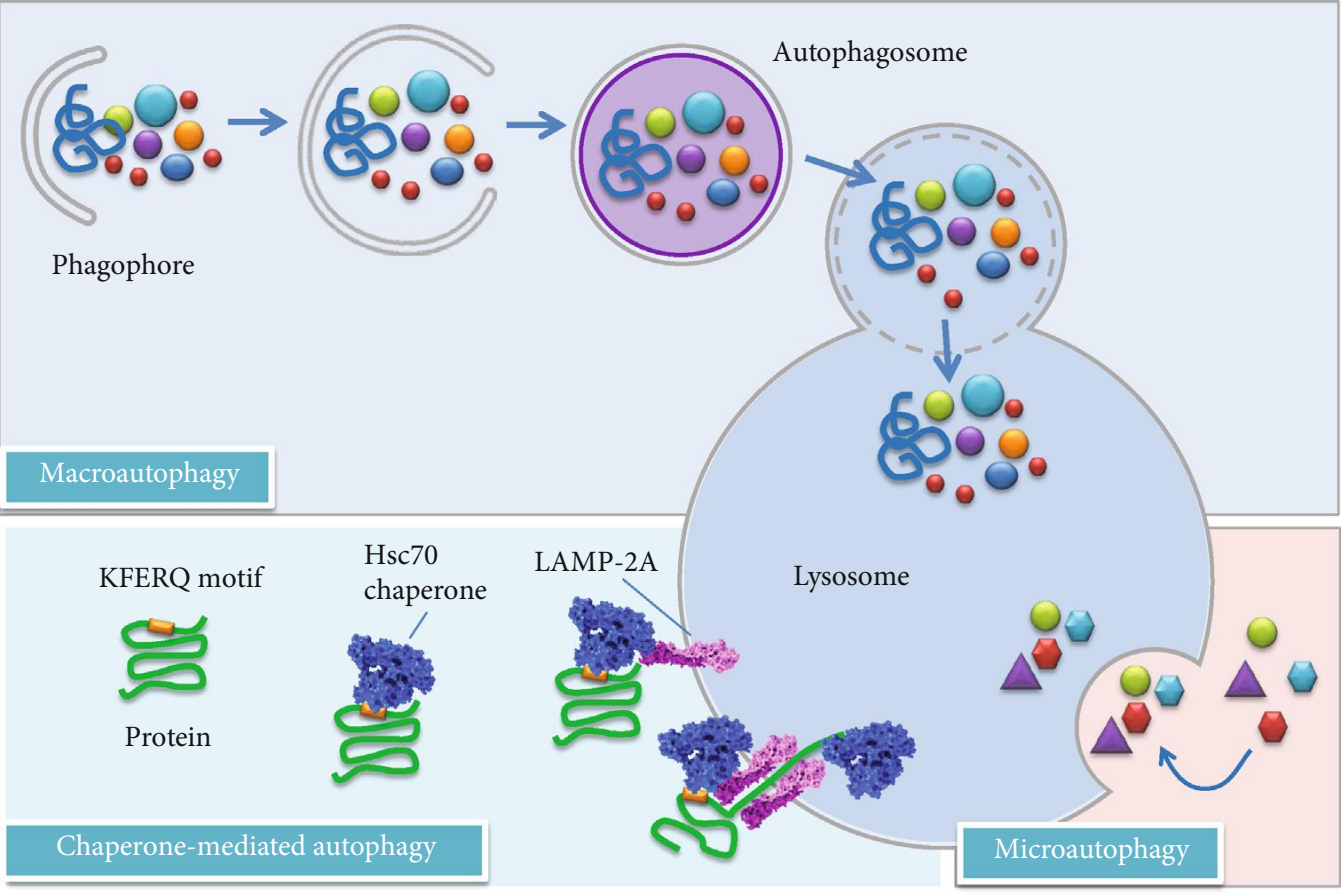
Different types of autophagy in mammalian cells. Macroautophagy requires sequential steps leading to the formation of autophagosomes. After the fusion of an autophagosome with a lysosome, the cargo is degraded and released to the cytosol. Chaperone-mediated autophagy involves the recognition of substrates (mainly abnormal or damaged proteins) with a KFERQ motif, whereas microautophagy involves direct sequestration of cytoplasmic portions into the lysosome. Modified according to [268].

Autophagy acts as a starvation response to maintain cellular nutrient levels, helping to regulate intracellular organelle homeostasis. It plays a crucial role in the removal of toxic/aggregated proteins and impaired organelles that could damage cells during stress, and its alteration is reported in various human pathologies, including neurodegenerative diseases, cancers, and lysosomal storage disorders [82, 83]. Autophagy occurs constitutively in all cell types at a basal level. The composition of the cytoplasm and the size of ER are thus regulated by autophagy [84]. Autophagy may also induce type II apoptosis, and its dysregulation is involved in the pathogenesis of several diseases. Autophagy is generally classified into three major types: macroautophagy, microautophagy, and chaperone-mediated autophagy (CMA) [see Figure 2]. All the three pathways deliver their cargo for lysosomal degradation but are mechanistically different from one another [85].

Macroautophagy (often indicated as autophagy) is the most extensively studied process that has an indispensable role in maintaining cellular and organismal homeostasis [86]. This evolutionarily conserved pathway involves a multitude of autophagy-related (Atg) proteins, coordinating vesicle formation throught three different steps (initiation, elongation, and maturation), followed by fusion with lysosomes to form the so-called autolysosomes [81]. Throughout macroautophagy superfluous organelles, pathogenic organisms, misfolded protein aggregates, and/or damaged mitochondria are sequestered into a double-membrane vesicle, known as autophagosome [87]. The initiation involves the formation of a phagophore, which is the primary double-membrane sequestering compartment in the cytoplasm. As the phagophore expands, the membrane curves to ultimately generate a spherical autophagosome, which then fuses with a lysosome to form an autolysosome, following the fusion of the outer membrane of the autophagosome with the lysosomal membrane. In this final step, the autophagosome cargo interacts with lysosomal hydrolases allowing its degradation. Exposed to the acidic lumen and resident hydrolases of the lysosome, the autophagosome inner membrane and, subsequently, the autophagic cargo are degraded. Then, the component parts are recycled back to the cytosol for fuelling other cellular pathways [88].

**Figure 3:**
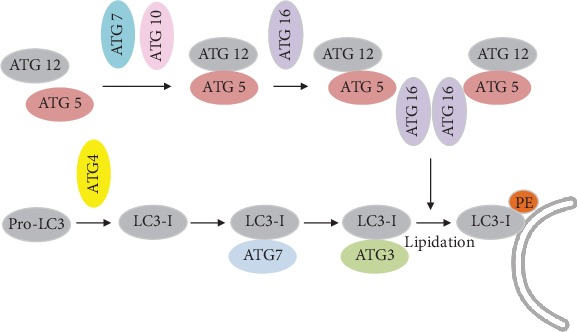
Schematic description of two ubiquitination-like modification systems essential for autophagy: Atg12-Atg5-conjugation and LC3-modification. Details can be found in the text.

The elongating step requires two processes comparable to ubiquitination: Atg12-Atg5-conjugation and LC3-modification [89, 90] (see Figure 3). In the first conjugation system (Atg12-Atg5 system), the ubiquitin-like protein Atg12 is activated by ATG7 and transferred to ATG10. The formation of this intermediate enables the conjugation of Atg12 to Atg5 followed by the formation of a noncovalent protein complex with Atg16 on the outside of the nascent autophagosome additionally promoting LC3I (microtubule-associated-protein-light-chain-3)-PE (phosphatidylethanolamine) formation analogous to E3 enzymes during ubiquitin conjugation reactions. In the second conjugation system, Atg4 eliminates the C-terminal tail of LC3 to expose a glycine residue (G116). Atg7 activates G116, followed by conjugated with Atg7. LC3I is then transferred to Atg3 and finally binds to the amino group of phosphatidylethanolamines (PE). This LC3-PE complex is called LC3II and localized at the autophagosomal membrane, enabling membrane elongation. Additionally, LC3II can bind to the adapter protein sequestosome 1 (SQSTM1/p62), playing a role in recognition and targeting of ubiquitinated protein aggregates to degradation via selective autophagy. Emerging evidence suggests that ubiquitin regulates the catabolism of cellular targets by both the UPS and autophagy system. Ubiquitin has seven lysine residues (K6, K11, K27, K29, K33, K48, and K63), each of which can participate in further ubiquitination. K48-linked polyubiquitin chains are recognized by the proteasomal system, while K63-linked chains or monoubiquitinated substrates are preferentially degraded by autophagosomal pathway [91]. Therefore, despite the use of ubiquitin in both pathways, the different structures of polyubiquitin chains may be adequate to maintain specificity and selectivity of autophagosomal and proteasomal pathways, but due to incomplete specificity of SQSTM1/p62, there is some overlap, even if the affinity of p62 for K63-linked polyubiquitin chains is higher [92]. Thus, recruitment into autophagosomes seems to be possible under circumstances when the UPS is compromised and act as an alternative mechanism (more details on crosstalk between autophagy and UPS can be found in [93] and in [94]). In addition, macroautophagy often converges with the endocytotic pathway. In this case, autophagosomes, before fusion with lysosomes, may also fuse with early or late endosomes to form amphisomes, which then fuse with lysosomes to become autolysosomes [95, 96].

In a different way, microautophagy involves the direct invagination of small parts at the lysosome surface, followed by vesicle degradation in the lysosomal lumen and liberation of breakdown products into the cytoplasm. This mechanism is only poorly understood [85, 97].

CMA [98] is a selective and direct elimination system, without building of a vesicle and highly selective for a particular group of soluble cytosolic proteins containing the pentapeptide KFERQ as recognition sequence. In brief, the motif consists of a glutamine (Q) flanked on either side by one or two of the positively charged residues K and R; one or two of the hydrophobic residues I, L, V, and F; and one of the negatively charged residues D and E [99]. Therefore, several different combinations of amino acids can result in a KFERQ-like motif within the sequence of a protein. It has been found that about 25-30% of cytosolic proteins contain a sequence motif related to the KFERQ motif [100, 101]. Uptake into the lysosome occurs via recognition by cytosolic chaperones and receptor-mediated translocation [102]. In a first step, the substrate binds to the cytosolic heat shock cognate protein of 70 kDa (hsc 70) and its co-chaperone, followed by binding to the lysosomal-associated membrane protein type 2A (LAMP-2A). After unfolding, the substrate protein is translocated into the lysosomal lumen with the assistance of a lysosomal chaperone (lys-hsc 70). It is assumed that several oxidative protein modifications and posttranslational modifications such as deamidation lead to the formation of a KFERQ-like motif and convert a non-CMA substrate into a CMA substrate which can be targeted for lysosomal degradation [103].

Overall, the high importance of the lysosomal system and the diversity and wealth of functions that can be assigned to autophagy explains the number of diseases in which autophagy deficiencies have been observed.

## 2. Proteostasis and Neurodegeneration

The integrity of the proteostasis network is essential to assure cell viability and is particularly important in nonproliferative cells, especially neurons, as its failure leads to a number of neurodegenerative diseases becoming prominent in the growing elderly population. Among these pathologies, Alzheimer's disease, Parkinson's disease, and amyotrophic lateral sclerosis are the most prevalent disorders that are characterized by the accumulation of misfolded proteins. Perturbations in the function of mitochondria, ER, and UPS/autophagy degradation pathways are emerging in these protein deposition diseases as relevant factors in driving synaptic dysfunction, alteration of axonal transport, and neuronal loss. Thus, the importance of correct protein folding and degradation systems in neurons is highly dependent on the proteostasis network supporting cell survival [104].

### 2.1. Alzheimer's Disease

*Alzheimer's disease* (*AD*) is an irreversible, progressive brain disorder slowly destroying cognitive function, resulting in memory loss, personality changes, psychosis, language disturbances, and eventually the complete loss of the ability to carry out the simplest tasks. AD is the most prevalent neurodegenerative disease, and the global number of people living with AD and other dementias has more than doubled from 1990 to 2016, mainly due to increases in population aging and growth [105]. There are two types of AD: familial early onset and sporadic late onset.

Familial AD is mainly genetically determined. Mutations in three causal genes, the *APP* gene on chromosome 21 [106], the *PSEN1* gene on chromosome 14 [107], and *PSEN2* gene on chromosome 1 [108], have been identified so far. Even though these mutations lead to early-onset AD and are mostly autosomal dominantly inherited, they represent only 5-10% of all early-onset cases. Indeed, over 50% of Mendelian cases and most of sporadic cases remain genetically unexplained [109–111].

Late-onset AD is considered to be multifactorial, although involving a strong genetic predisposition. A genome-wide association study confirmed that the *ε*4 allele of *APOE* is the strongest genetic risk factor for AD, associated with increased risk for both early- and late-onset AD. Furthermore, there is strong evidence that obesity, diabetes, cardiovascular disease, and systemic inflammation contribute to the risk for late-onset AD and decrease the age at which symptoms appear first [112–114].

Neuropathologically, AD is characterized by an extensive extracellular deposition of Amyloid *β* (A*β*) peptides in amyloid plaques and can be considered to be a localized form of brain amyloid disease (amyloidosis). An additional disease hallmark is the presence of intracellular neurofibrillary tangles (NFTs) of hyperphosphorylated Tau protein [115]. A*β* is a 39-43 amino acid peptide that becomes misfolded and self-associates to form extensive amyloid deposits in AD patients. A*β*40 is the major A*β* species, whereas A*β*42 is a minor species aggregating much more readily *in vitro* into amyloid fibrils and is heavily enriched in senile plaques [116]. A*β* is generated from the *β*-amyloid precursor protein (APP) with alternative splicing of APP mRNA leading to multiple different length isoforms of APP. The human APP gene is located on chromosome 21 with three major isoforms arising from alternative splicing [117], namely, APP_695_, APP_751_, and APP_770_ (with 695, 751, and 770 referring to the number of amino acids), all of which can generate A*β* after sequential cleavages by *β*- and *γ*-secretase [118, 119]. There are two divergent pathways (in detail reviewed in [120]), the minor of which is amyloidogenic and involves the action of two membrane-bound proteases termed *β*- and *γ*-secretases. The major pathway with more than 90% of APP metabolism, however, normally involves the initial alternative cleavage of APP by a zinc metalloprotease termed *α*-secretase followed again by *γ*-secretase. *α*-Secretase cleaves APP within the A*β* peptide region, therefore preventing A*β* formation.

Full-length APP is synthesized in the ER and transported through the Golgi/trans-Golgi network (TGN) apparatus, where APP undergoes posttranslational modifications during maturation [121]. Full-length APP, *α*-, *β*-, and *γ*-secretase are transported to the cell surface in TGN-derived secretory vesicles. At the plasma membrane, APP is mainly cleaved by *α*-secretase to produce non-amyloidogenic sAPP*α* [122]. Uncleaved APP and secretases at the plasma membrane are reinternalized to the endosome and may either be recycled back to cell surface or undergo degradation after delivery to the lysosmal system. Elevated retention of APP in endosomes/lysosomes promotes amyloidogenic processing and A*β* production, due to optimal acidic environment for *β*-secretase activity [123]. Following its formation, A*β* can be degraded by proteolysis or cleared from the brain into the peripheral blood circulation. Neprilysin, insulin-degrading enzyme (IDE), endothelin-converting enzyme (ECE), and angiotensin-converting enzyme (ACE) have been reported to be capable of degrading A*β* [124, 125]. Additionally, the UPS serves as a major regulator of A*β* accumulation in neuronal cells, either by decreasing the production of A*β* or promoting its proteolytic degradation [126]. Therefore, any dysregulation in the UPS may also lead to A*β* accumulation in the cytoplasm of neurons, facilitating the formation of A*β* plaque [127]. Furthermore, the proteostatic regulatory mechanisms in the ER, such as ERAD and UPR, are commonly dysregulated in neurodegenerative diseases. It was recently shown that the ER protein membralin, an essential component of the ERAD complex mediating the degradation of ER luminal and membrane substrates, is downregulated in AD, suggesting a critical role for ERAD in AD pathogenesis [128]. The amyloid cascade hypothesis [129] has continued to gain support over the last two decades, although some revisions have been made in the last years [130–132]. In particular, emphasis has shifted away from amyloid fibrils as the predominant toxic form of A*β* and towards smaller and soluble oligomers or protofibrils [133, 134]. This rethinking has been prompted to some extent by the finding that the relationship between the numbers of senile plaques and the severity of dementia is poor and that small soluble oligomers seem to be more toxic than fibrillar A*β*. The second protein associated with AD is tau, a structural protein associated with microtubules playing a role in axonal transport [135, 136]. Hyperphosphorylated, insoluble, filamentous tau was shown to be the main component of NFTs found in AD [137]. Furthermore, tau inclusions are thought to contribute to AD pathogenesis due to their occurrence in brain regions with altered function, and NFT formation correlates with the duration and progression of this disease [138]. Tau is an unstructured protein, and *in vitro* degradation experiments showed that tau can be degraded by the 20S without ubiquitination [37]. Tau is expressed in the central and peripheral nervous system and in kidney, lung, and testis, even if to a lesser extent [139]. Most abundantly, it is found in neuronal axons [140] and in oligodendrocytes [141] and neuronal somatodendritic compartments [142]. Investigations on tau turnover indicate that both autophagy and UPS seem to play a role in its degradation [143, 144], with preferences for distinct pools. For example, hypoacetylated tau is preferentially degraded by UPS [144, 145]. The accumulation of advanced glycation end products (AGEs) has also been identified as a hallmark of AD [146]. AGEs form insoluble protein aggregates associated with increased oxidative stress and inflammation [147]. It was demonstrated that AGEs increase ROS production, stimulating downstream pathways related to APP processing and A*β* production [148]. Additionally, increased expression of the immunoproteasome has been identified within amyloid plaques [54], the primary location of AGEs. It was already shown that accumulation of AGEs results in a robust increase in immunoproteasome expression and activity via activation of the AGE-receptor (RAGE) and triggers phosphorylation of STAT1 via Jak1/2, resulting in the transcriptional increase of immunoproteasome subunits [29].

### 2.2. Parkinson's Disease

Parkinson's disease (PD) is an age-related deterioration of dopaminergic neurons in the *substantia nigra* and other brain regions. The pathological hallmark of PD is the cytoplasmic deposition of amyloid-like aggregates termed Lewy bodies, the fibrous inclusions which are predominantly composed of *α*-synuclein (*α*S) protein [149, 150]. As in other neurodegenerative disorders, most of the experimental evidence points to protein aggregation as a gain-of-toxic-function process that plays a central role in the pathogenesis [15, 151], although the detailed nature of the toxic species remains to be established. Growing experimental evidence suggests that specific oligomeric species are the most cytotoxic forms of *α*S and are likely to play a key role in disease [152–156], although some studies have shown that *α*S fibrils can also be highly toxic [157, 158]. Nevertheless, the etiology of PD remains largely unknown, and it is actually considered to be sporadic in about 90% of cases, with 10-15% of patients having a positive family history of PD [159]. The most relevant risk factor for PD is aging, as the incidence increases nearly exponentially with age and joins a peak after 80 years [160]. However, in the last two decades, it becomes clear that the exposure to different environmental factors could strongly contribute to the late-onset sporadic PD cases, alone or in combination with genetic factors. Autosomal recessive forms of PD are associated with mutations to *Parkin*, *PINK1*, and *DJ-1* genes [161]. Unlike autosomal dominant PD, whose age of onset is quite similar to the sporadic one, monogenic forms of recessive Parkinsonism are an important cause of early-onset pathology (before 40 years of age) [162].

Parkin protein is an E3 ubiquitin ligase, so it is involved in the degradation of targeted proteins through UPS. Mutations in the Parkin encoding gene have been associated with ubiquitin ligase-activity dysfunction, finally resulting in accumulation of proteins that cause neuronal toxicity, particularly in the *substantia nigra* [163]. A wide range of studies supported this hypothesis, demonstrating that *α*S is degraded by the proteasome with both ubiquitin-dependent [164–166] and ubiquitin-independent [167, 168] mechanisms. In addition, the largest amount of *α*S present in Lewy bodies is phosphorylated at residue S129 (pS129), and the inhibition of UPS by MG132 or lactacystin was found to cause a significant accumulation of pS129 in human neuroblastoma cells, without affecting the total amount of protein. Considering these evidences, it has been hypothesized a role for this posttranslational modification in targeting the protein to the proteasome pathway, where a rapid degradation occurs through a ubiquitin-independent mechanism [169]. However, *α*S is predominantly degraded by lysosomes. Indeed, *PINK1* encodes the PTEN-induced putative kinase 1, a serine-threonine protein kinase that was found to interact with Parkin promoting the selective autophagy of damaged mitochondria [170]. The autophagic pathway is implicated in the degradation of both cytosolic and vesicular *α*S. In particular, misfodeld *α*S aggregates strongly colocalize with both early and late endosomal/lysosomal markers [171–173], thus indicating that *α*S is subjected to lysosomal degradation, even if there are some conflicting reports about the kinetics of this process, possibly because of differences in the experimental procedures applied for the preparation of the aggregates and their delivery to cells [140]. Vesicles or multivesicular bodies containing *α*S can also be targeted for exocytosis to unburden the lysosomal degradation pathway, causing the release of pathogenic *α*S from the neuron. The involvement of autophagy in *α*S clearance has also been demonstrated *in vitro* by using 3-methyladenine (3-MA), a selective autophagy inhibitor, that caused a significant increase of both soluble and aggregated *α*S levels in nonneuronal cells [174]. Furthermore, the accumulation of A53T oligomers was observed in neuroblastoma cells following both pharmacological and molecular inhibition of the macroautophagic process [175]. However, there are some conflicting works reporting that the 3-MA inhibition did not result in any accumulation of insoluble *α*S, both in neuronal cultured cells and in animal models, suggesting that another lysosomal degradation mechanism probably occurs in *α*S clearance [176]. *α*S overexpression significantly impaired macroautophagy both in mammalian nonneuronal cells and in transgenic mouse models, by inhibiting Rab1a protein, a key early mediator of the process, causing the mislocalization of the autophagy protein Atg9 and the decrease in autophagosome maturation [177]. Macroautophagy has been proposed as the elective mechanism for the degradation of fibrillar assemblies, whereas oligomeric species are more likely degraded by CMA. Indeed, CMA showed a crucial role in the regulation of intracellular *α*S levels [178]. A wide range of studies proved the relevance of this process in the degradation of monomeric and dimeric *α*S in isolated lysosomes *in vivo* and that A30P and A53T *α*S mutations strongly inhibit CMA [179]. Furthermore, the downregulation of *LAMP-*2A, the rate-limiting step in the CMA pathway, was found to cause a significant increase of aggregated *α*S, suggesting its central role in the degradation of *α*S [174].

Through lysosomal rupture, a small amount of misfolded *α*S can escape the degradation pathway, thus becoming free in the cytosol [180]. In addition, the use of chloroquine, a lysosomal inhibitor, leads to a substantial increase of cytosolic misfolded *α*S [181]. Importantly, lysosomal functionality is found to be dramatically impaired with age, and this could be one of the most relevant mechanisms by which age could contribute to *α*S pathology [148, 180]. As a consequence, failures in the clearance of *α*S are responsible for the accumulation of toxic aggregates, as shown both in cultured cells and in animal models [182, 183]. Once inside the cells, *α*S forms perinuclear thread-like inclusions, further subjected to many modifications, such as phosphorylation and ubiquitination [184]; studies performed in mice brains indicated that these pools of pathogenic *α*S can persist for months [185], but small oligomeric assemblies, derived from these large inclusions, could be released by the cells.

Finally, *α*S degradation can also occur through the activation of other proteases, such as calpain [186], neurosin (kallikrein-6) [187], and matrix metalloproteinases [188], both *in vitro* and *in vivo*. The products of such cleavage are truncated forms of the protein with the ability to induce the aggregation of the full-length endogenous protein, and together with aggregated *α*S, they were found to be components of Lewy bodies and neurites [189].

### 2.3. Amyotrophic Lateral Sclerosis

Amyotrophic lateral sclerosis (ALS) is a devastating neurodegenerative disease characterized by a progressive loss of motor neurons of the central nervous system (CNS) leading to muscle weakness, wasting, and spasticity. In 2006, the predominately nuclear TAR DNA-binding protein 43 (TDP-43) was identified as a key component of the insoluble and ubiquitinated inclusions in the brains of patients suffering from amyotrophic lateral sclerosis (ALS) and frontotemporal lobar degeneration (FTLD or FTLD-TDP) diseases [190, 191]. The cytoplasmic deposition of TDP-43 occurs concomitantly with the depletion of native TDP-43 from the nucleus [191], causing neurodegeneration by a combination of gain-of-function (GOF) and loss-of-function (LOF) mechanisms [191–193]. Cytosolic aggregates are known to be intrinsically toxic [192, 194] and able to recruit nuclear TDP-43, improving their deleterious effects by contributing to the nuclear LOF [195–197]. The proteostasis systems appear to be impaired in ALS, as well as other physiological functions [198], suggesting that it is crucial for neurons to maintain TDP-43 protein homeostasis by the protein degradation pathways [145, 199–201]. Indeed, a progressive decrease in the efficiency of cellular proteostasis has been reported as an important factor contributing to ALS onset in elderly people [198]. This hypothesis is also supported by genetic evidence, as many of the mutations associated with ALS affect genes involved in UPS- or autophagy-mediated degradation, such as VCP, CHMP2B, progranulin (PGRN), OPTN, TMEM106B, SQSTM1, TBK1, UBQLN2, ALS2, and C9ORF72 [86, 126, 202–210].

Recent studies showed that cytosolic accumulation of TDP-43 is turned over mainly by UPS, even if ALP contributes to the degradation TDP-43 intractable aggregates [200, 211]. Accordingly, cytosolic diffuse TDP-43 was found to be assembled in different misfolded species, ranging from soluble monomer to undegradable macroaggregates [211], even though a pool of TDP-43 inclusions cannot be degraded [200, 211, 212]. In particular, we have recently reported that, among the various TDP-43 aggregates distinguishable on the basis of their susceptibility to the different protein degradation systems, the undisposable fraction corresponds to *ca*. 49%, whereas those degradable via UPS and ALP account for *ca*. 24% and 26%, respectively [212]. These data indicate that both routes contribute equally to the degradation of TDP-43 aggregates. However, the UPS- and ALP-mediated degradation processes appear to be kinetically independent, acting on different populations of TDP-43 aggregates that are not in equilibrium or interconvert on a slow time scale [212]. Indeed, the inhibition of one system caused an increase of the degradation rate, even if the amount of degraded TDP-43 is lower, with respect to the condition in which both routes are at work. These findings have confirmed previous observations that the inhibition of one clearance pathway renders the remaining one more effective [213, 214] and reinforces the view that a crosstalk exists between the two clearance systems.

Ubiquitination of TDP-43 is one of the major features of pathological TDP-43 that has been found in the brain and spinal cord of patients with ALS [155]. Thus, the pathogenic mechanism of ubiquitinated TDP-43 in ALS, including the origin and redistribution of pathological TDP-43, has been studied intensively in the past ten years. The E3 ubiquitin ligase (Parkin) is shown to ubiquitinate TDP-43 via the ubiquitin lysines, K-48, and K-63. This facilitates the TDP-43's cytoplasmic accumulation into inclusions without any detectable evidence of its protein degradation [215, 216]. Other studies have also shown that TDP-43 aggregates formed from the full-length protein are labeled by both K-48 and K-63-linked polyubiquitin chains and are directed toward different fates: UPS degradation of TDP-43 for the K-48-linked polyubiquitin chains and autophagic removal of the TDP-43 with K-63-linked polyubiquitin chains [200]. In a yeast two-hybrid screen, the ubiquitin-conjugating enzyme UBE2E3 and the ubiquitin-isopeptidase Y (UBPY) were identified to interact with TDP-43, enhancing the ubiquitination and accumulation of its insoluble high molecular weight aggregates [98]. Furthermore, mutations at the ubiquitination sites in TDP-43 were also found to decrease its accumulation thus implicating the ubiquitination in modulating the TDP-43 aggregation [217]. In particular, an FTLD-associated TDP-43 with K263E mutation was observed to be excessively ubiquitinated, possibly as a consequence of its misfolding due to the substitution of the positively charged lysine residue with a negatively charged aspartate residue in the RRM2 domain [98]. Additionally, mutations at the ubiquitination sites near the TDP-43's RRM1 domain were also found to decrease the TDP-43's accumulation, thereby implicating the ubiquitination in modulating the TDP-43 aggregation [217].

The role of autophagy in rescuing TDP-43-associated toxicity might be a complex process as suggested by conflicting data showing that autophagy can either accelerate or slow down disease progression [199]. Indeed, the vacuolar fusion machinery and the endo-lysosomal pathways have been found to be critical for the TDP-43 clearance and for maintaining the cell survival. Defective endocytosis might be a related factor for the TDP-43-related toxicity in ALS. In particular, abnormal levels of TDP-43 have been found to prevent endocytosis by colocalizing with the endocytosis-associated proteins, both in yeast and cellular models and in the frontal cortex tissue of an ALS patient [218]. Impaired endocytosis has been linked with an increase in TDP-43 aggregation, whereas enhancing endocytosis can reverse the TDP-43 toxicity and the motor neuron dysfunction [218]. Another study has also shown that the endocytosis and the endo-lysosomal pathway are markedly disturbed by TDP-43 expression that probably alters the expression of key endocytic components [102].

## 3. Oxidative Damage to Proteostasis Network in Neurodegenerative Diseases

**Figure 4:**
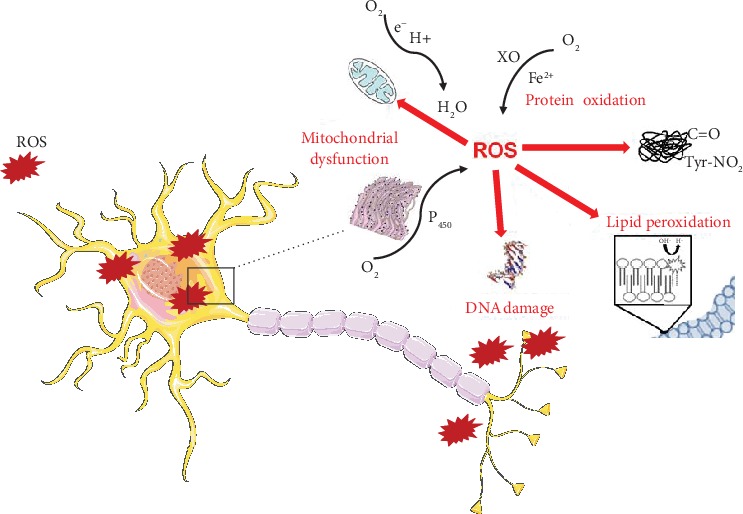
Cellular damage induced by oxidative stress: protein oxidation, lipid peroxidation, DNA damage, mitochondrial dysfunction, and perturbation of the endoplasmic reticulum. Cell membrane illustration was used from Servier Medical Art under creative commons license 3.0 (https://smart.servier.com/).

The central nervous system (CNS) is particularly vulnerable to oxidative stress (OS), suggesting its important role in the pathogenesis of neurodegenerative diseases. Increasing numbers of individuals, particularly the elderly, suffer from neurodegenerative disorders. These diseases are normally characterized by progressive loss of neuron cells and compromised motor or cognitive function. Previous studies have proposed that the overproduction of reactive oxygen species and nitrogen species (ROS/RNS) may have complex roles in promoting the disease development [219, 220]. Research has shown that neuron cells are particularly vulnerable to oxidative damage due to their high polyunsaturated fatty acid content in membranes, high oxygen consumption, and weak antioxidant defense [219]. However, the exact molecular pathogenesis of neurodegeneration related to the disturbance of redox balance remains unclear. Several lines of evidence support the notion that OS play a detrimental role in the pathogenesis of neurodegenerative disorders, causing the damage of vital cellular elements such as nucleic acids, lipids, and proteins [220]. OS refers to a condition where ROS/RNS production overwhelms the cellular antioxidant defense system. Indeed, antioxidant defenses are insufficient to keep the levels of ROS/RNS below a toxic threshold. This may be either due to excessive production of ROS, loss of antioxidant defenses, or both. Glutathione (GSH) is an important intracellular antioxidant that protects against a variety of different antioxidant species. In addition, an age-related decline in GSH has been observed in a number of senescent organisms including mosquitoes, fruit flies, mice, rats, and humans [221, 222]. GSH is, also, considered the major thiol redox buffer to maintain intracellular redox homeostasis. On the other hand, the formation of ROS/RNS is strongly exacerbated in the aging process, thus affecting the ability of the cells to maintain their proteostasis, although the exact mechanisms are still discussed. ROS/RNS include both radical and nonradical oxygen species formed by the partial reduction of oxygen such as superoxide radical anion (O2− •), hydrogen peroxide (H_2_O_2_), hydroxyl radical (HO•), nitric oxide (• NO), and peroxynitrite (ONOO−). The principal source of ROS in the cell is the mitochondrial respiratory chain. Indeed, mitochondrial complexes I and III can leak electrons causing the partial reduction of oxygen to O2⨪. The resulting O2⨪ can spontaneously, or by the superoxide dismutase (SOD)-mediated catalysis, disproportionate very rapidly into H_2_O_2_. The antioxidant enzymes SOD, catalase, and glutathione peroxidases (GSH-Px or GPx) are responsible for removal of O2⨪, H_2_O_2_, and peroxides in general [223]. Many lines of evidence were collected, indicating that ROS and RNS were able to modify proteins in a reversible manner. Indeed, OS results from the accumulation of oxidized and damaged macromolecules that are not efficiently removed and renewed (Figure 4). Proteins are highly susceptible to oxidative damage that inevitably affects secondary and tertiary structure, resulting in irreversible modification of protein structure and function. There are four major indices of protein oxidation: protein carbonylation, protein nitration, protein bound-lipid peroxidation products, and protein glycoxidation (Figure 3). Protein carbonyl groups are generated from the direct oxidation of various side chains of amino acid residues (Lys, Arg, Pro, Thr, His, and others) by the removal of hydrogen atoms from the alpha carbons and by the cleavage of the peptide chain [224]. Protein tyrosine nitration (3-NT) is considered an irreversible form of protein damage and therefore a robust biomarker of nitrosative stress [225, 226]. Intriguingly, the addition of a large nitro group in the 3′ position causes steric constraints on the tyrosine residue, which, in turn, affects its phosphorylation, via tyrosine kinases, and therefore can compromise several signaling mechanisms [227].

The brain has high concentrations of polyunsaturated fatty acids (PUFAs), such as docosahexaenoic acid and arachidonic acid, compared with other organs. As these fatty acids are highly unsaturated, OS makes them susceptible to lipid peroxidation, which is one of the major outcomes of free radical-mediated injury (Figure 3) [228]. PUFAs of neuronal membranes can be broken down causing the release of elevated levels of reactive electrophilic aldehydes, which covalently bind proteins thus forming several adducts [229], such as 4-hydroxynonenal (4-HNE). Several investigations demonstrated increased brain lipid peroxidation and nitrosative stress in the brain of AD and PD patients [230–232].

Many studies have shown links between protein homeostasis and OS. Proteins that are irreversibly oxidized need to be degraded by the proteostasis network [27, 229], but the capacity of cells to maintain proteostasis decreases during aging, leaving the organism susceptible to neurodegeneration. In light of these findings, the major consequence of protein oxidation is the formation of large protein aggregates, whose accumulation becomes toxic to cells. Insoluble aggregates can be formed as a result of covalent cross-links among peptide chains, as in the case of A*β* peptide and NFTs in AD, *α*-synuclein in PD, and mSOD1 in ALS. Indeed, OS enhances *γ*-secretase and *β*-secretase (BACE-1) activity and A*β* production in neurons. Studies conducted by Tamagno et al. showed that OS induces an increase of BACE protein levels and activity, suggesting a direct relationship between OS and the amyloidogenic processing of APP [233, 234]. In particular, exposure of NT2 neurons to 4-HNE caused a significant increase of intracellular and extracellular levels of both A*β*-40 and A*β*-42 species and an overproduction of A*β* through the overexpression of BACE-1 [234]. In addition, immunohistochemical studies demonstrated that 4-HNE and 3-NT modifications are also present in NFTs in AD [235]. These results supported that NFTs are one of the principal loci for protein oxidation in AD. Considering the involvement of A*β* in oxidative processes, the methionine (met) at residue 35 is one of the most intriguing amino acid residues in the peptide molecule, as it has the most easily oxidized side chain in the peptide, and it is partially oxidized in postmortem amyloid plaques [236, 237], contributing to the oxidative damage of A*β* (1−42) [238, 239]. Butterfield's group demonstrated that substitution of Cys for Met in human A*β* (1–42) resulted in no protein oxidation in a *C. elegans* model [240]. Met may have several important cellular functions [241]; among them, it protects the active site of enzymes against oxidation. Indeed, methionine sulfoxide reductase (MSR) activity, that catalyzes the conversion of Met-sulfoxide (MetSOx) to Met, is reduced in AD brain, which may lead to increase oxidation of Met [242].

Recently, lipid peroxidation and nitrosative stress started to be considered potential promising biomarkers of AD. However, little and controversial knowledge has emerged about the antioxidant functionality of the heme oxygenase-1/biliverdin reductase-A (HO-1/BVR-A) system in blood. Di Domenico *et al*. described that the HO-1/BVR-A system status in plasma might reflect the ongoing situation in the brain, offering an important biochemical tool for the potential prediction of AD at the earliest stages of the disease [243]. In addition, several studies measured 4-HNE in serum or CSF of AD patients, and they start to correlate the higher levels of 4-HNE and 3-NT to the degree of cognitive impairment [244, 245]. Moreover, MDA and 4-HNE were found higher in fibroblasts and lymphocytes from AD patients with respect to controls [245]. This line of research tried to associate the 4-HNE and 3-NT modifications with proteins present in cells, such as lymphocytes, which can be collected in antemortem patients to study the progression of AD [246]. Anyway, although these and other several evidences support that peripheral cells recapitulate major molecular mechanisms affected in the brain, further analyses are required as these dividing cells differ in mechanisms essential for the development of AD.

OS plays an important role in the degeneration of dopaminergic neurons in PD, and it has been linked to both sporadic and familial forms of the disease. In particular, OS was found to alter dopamine transport contributing to its loss. *α*-Synuclein itself contributes to OS through the decrease of mitochondrial function and the increase of ROS production in transgenic mice and various cell models [247, 248]. The link between OS and dopaminergic neuronal degeneration is further supported by the identification of decreased levels of glutathione, the major reducing agent in the cell, in the *substantia nigra* of PD brains [249]. Dexter et al. demonstrated the involvement of lipid peroxidation in PD as causative agent of nigral cell death [250]. Subsequent studies showed that 3-NT modifications of *α*-synuclein can lead to a neurotoxic form of the protein able to kill dopaminergic neurons [251]. Moreover, studies investigating lipid peroxidation in the CSF of PD patients showed increased levels of 4-HNE [252]. In a study of Di Domenico et al., *C. elegans* expressing mutant LRRK2, the most common causative gene of autosomal-dominantly inherited and idiopathic PD, showed increased protein oxidation and lipid peroxidation [253].

Increased markers of OS have been found in spinal cord, motor cortex, cerebrospinal fluid, or serum of sporadic ALS patients compared with healthy subjects [254]. Although the exact causes of motor neuron degeneration in sporadic ALS remain unclear, increased OS is reported as a relevant factor in the pathogenesis of the disease. In addition, about 20% of persons with inherited ALS have a mutation in the antioxidant enzyme SOD1, which leads to cellular toxicity [255]. The oligomerization hypothesis states that mutant SOD1 proteins are prone to misfold generating deleterious intracellular aggregates that can alter cellular homeostasis and reduce the availability of essential proteins for normal cellular function [256].

## 4. Crosstalk between Autophagy and Oxidative Stress in Neurodegeneration

Autophagy is activated under stress conditions such as starvation, ischemia/reperfusion, and pathogen infection and is deregulated in various pathological conditions, including cancer and neurodegenerative diseases. It is generally accepted that ROS induce autophagy and that autophagy, in turn, serves to reduce oxidative damage [257]. Indeed, it has been shown that low amounts of ROS can activate the adaptive cellular apparatus to increase the organism's stress resistance. This involves the enhancement of antioxidant and heat shock responses, cell cycle regulation, apoptosis, DNA repair, UPR, and autophagy. Conversely, a chronic exposure to ROS can induce oxidation of proteins as well as specific component of ER, UPS, or autophagy pathways, thus exacerbating the accumulation of unfolded/misfolded proteins.

These findings suggest that autophagy plays a crucial role in maintaining the redox status as OS and reactive oxygen/nitrogen species can induce autophagy, and the antioxidant treatments can inhibit autophagy. Both O^2-^ and H_2_O_2_ have been shown to affect autophagy downstream of nutrient starvation. There is likely a tight regulatory loop involving redox signaling, mitochondria, and mitophagy (the selective form of autophagy that degrades damaged mitochondria) as the mitochondria are thought to be the main sources of these autophagy-inducing ROS. Indeed, healthy mitochondria produce ROS as byproducts of oxidative phosphorylation; however, damaged mitochondria can generate copious amounts of ROS while simultaneously signaling their own self-removal via mitophagy, forming a redox signal negative feedback loop. Acute oxidative stress can lead to rapid upregulation of autophagy via posttranslational modifications of key autophagy regulators. Increased production of ROS stimulates the initiation of autophagy in association with stress signal pathways. For this, cysteine protease Atg-4 inactivation is made with ROS accumulation in the cell, resulting in Atg-4 phosphoethanolamine precursor accumulation, which is also necessary for the beginning of autophagosome [258]. Sustained or chronic oxidative stress can also lead to transcriptional activation of CMA via LAMP2A.

The effects of deficient proteostasis network and increased oxidized proteins can together induce a vicious cycle triggering neurodegeneration [83, 259]. A recent study demonstrated the impairment of autophagy and its related pathway in AD human brain [260]. Autophagy was also implicated in the degradation of *α*-synuclein. Indeed, converging evidences from genetic, pathological, and experimental studies have provided indications that the autophagic pathway is compromised in PD [261]. In transgenic mouse models of AD and AD-like dementia, the restoring of the autophagic deficits led to a reduction of OS and A*β* brain deposition and prevented learning and memory deficits [262, 263]. A reduction in *α*-synuclein aggregation and mitochondrial dysfunction has also been found in animal models of PD whose autophagy system had been increased [264]. Previous reports have shown that autophagy can degrade mutant SOD1, preventing its cytoplasmic accumulation and toxicity. In addition, the inhibition of motor neuron autophagy in SOD1-G93A mice was found to induce neuromuscular denervation in the early stages of the disease. A recent study demonstrates that rapamycin, a selective mTOR inhibitor, provides behavioral improvement and reduces the loss of dopaminergic neurons in PD. The neuroprotective properties of rapamycin arise from its capacity to reduce OS and mitochondrial injuries [265]. Another recent article has shown that bosutinib, a SRC inhibitor which boosts autophagy, can improve the survival of ALS iPSC-derived motor neurons from patients with familial ALS patients carrying SOD1 mutations [266]. Taken together, these findings suggest that pharmacologic interventions and/or lifestyle alterations which increase brain autophagy and proteostasis might be useful for the treatment and the prevention of age-associated neurodegenerative disorders.

## 5. Conclusions

Proteins play crucial roles within the cell, exerting their biological function following the acquisition of an appropriate three-dimensional conformation. Once the proteins have correctly folded and their function is fulfilled, they need to be properly degraded avoiding any possible cellular damage. In addition, unnecessary proteins or those synthesized in excess, that hamper cellular homeostasis, should be discarded rapidly. Thus, the prompt elimination of impaired proteins is essential for cell viability. The decrease in the efficiency of the clearance systems has been reported in the context of aging and neurodegenerative diseases, such as AD, PD, and ALS. Furthermore, the presence of gene mutations associated with cellular proteostasis in the familial forms of these neurodegenerative diseases suggest that a failure of the protein degradation systems strongly contributes to their etiology. In this scenario, several studies have reported that restoring the impaired degradation systems can improve cell survival. Nevertheless, further elucidations on the individual steps of proteostasis and its role in the pathogenic mechanisms will provide a better understanding of this important cellular process, aiming to develop new pharmacological treatments of aging-related and oxidative stress-related human diseases.
